# A Joint Modeling Approach for Childhood Meat, Fish and Egg Consumption and the Risk of Advanced Islet Autoimmunity

**DOI:** 10.1038/s41598-019-44196-1

**Published:** 2019-05-23

**Authors:** Essi Syrjälä, Jaakko Nevalainen, Jaakko Peltonen, Hanna-Mari Takkinen, Leena Hakola, Mari Åkerlund, Riitta Veijola, Jorma Ilonen, Jorma Toppari, Mikael Knip, Suvi M. Virtanen

**Affiliations:** 1 Health Sciences/Faculty of Social Sciences, Tampere University, Tampere, FI-33014 Finland; 2 Faculty of Information Technology and Communication Sciences, Tampere University, Tampere, FI-33014 Finland; 3 Department of Public Health Solutions, National Institute for Health and Welfare, Helsinki, FI-00271 Finland; 4 Department of Pediatrics, Medical Research Center, PEDEGO Research Unit, Oulu University Hospital and University of Oulu, Oulu, FI-90014 Finland; 5 Immunogenetics Laboratory, Institute of Biomedicine, University of Turku, Turku, FI-20520 Finland; 6 Department of Clinical Microbiology, Turku University Hospital, Turku, FI-20520 Finland; 7 Department of Pediatrics, Turku University Hospital, Turku, FI-20521 Finland; 8 Department of Physiology, Institute of Biomedicine, University of Turku, Turku, FI-20520 Finland; 9 Children’s Hospital, University of Helsinki and Helsinki University Hospital, Helsinki, FI-00281 Finland; 10 Research Programs Unit - Diabetes and Obesity, University of Helsinki, Helsinki, FI-00290 Finland; 11 Tampere Center for Child Health Research, Tampere University Hospital, Tampere, FI-33521 Finland; 12 Folkhälsan Research Center, Helsinki, FI-00290 Finland; 13 Tampere University Hospital, Research, Development and Innovation Center, Tampere, FI-33521 Finland; 14 Center for Child Health Research, Tampere University and Tampere University Hospital, Tampere, FI-33014 Finland

**Keywords:** Type 1 diabetes, Risk factors

## Abstract

Several dietary factors have been suspected to play a role in the development of advanced islet autoimmunity (IA) and/or type 1 diabetes (T1D), but the evidence is fragmentary. A prospective population-based cohort of 6081 Finnish newborn infants with *HLA-DQB1*-conferred susceptibility to T1D was followed up to 15 years of age. Diabetes-associated autoantibodies and diet were assessed at 3- to 12-month intervals. We aimed to study the association between consumption of selected foods and the development of advanced IA longitudinally with Cox regression models (CRM), basic joint models (JM) and joint latent class mixed models (JLCMM). The associations of these foods to T1D risk were also studied to investigate consistency between alternative endpoints. The JM showed a marginal association between meat consumption and advanced IA: the hazard ratio adjusted for selected confounding factors was 1.06 (95% CI: 1.00, 1.12). The JLCMM identified two classes in the consumption trajectories of fish and a marginal protective association for high consumers compared to low consumers: the adjusted hazard ratio was 0.68 (0.44, 1.05). Similar findings were obtained for T1D risk with adjusted hazard ratios of 1.13 (1.02, 1.24) for meat and 0.45 (0.23, 0.86) for fish consumption. Estimates from the CRMs were closer to unity and CIs were narrower compared to the JMs. Findings indicate that intake of meat might be directly and fish inversely associated with the development of advanced IA and T1D, and that disease hazards in longitudinal nutritional epidemiology are more appropriately modeled by joint models than with naive approaches.

## Introduction

Finland has the highest incidence of type 1 diabetes (T1D) in the world in children aged less than 15 years with the average incidence of 62.5 per 100,000 person-years during 2006–2011^1^. The incidence doubled during 1980–2005^2^ but reasons for the increase remain unclear. The highest increase in incidence rates were seen among the youngest children^3^, which could imply the role of early exposures in tahe disease process. Although infant-feeding patterns in relation to T1D have been studied broadly^4,5^, including the finding of no T1D-protective effect of weaning to hydrolyzed infant formula compared to a conventional cow’ s milk based formula in a large randomized controlled trial^4^, knowledge of food consumption during childhood and the risk of T1D is limited and studies with longitudinal exposure assessment are scarce^5^.

Longitudinal associations between exposures and disease are often investigated with a Cox regression model (CRM) including longitudinal measurements as a time-dependent covariate, which is defined as for example, a step function of last recorded values^6^. The associated crude approximation and disregard of measurement error and/or within individual variation can lead to biased estimates and standard errors^7^. Joint models for longitudinal and time-to-event data^7–9^ have been developed to address this challenge. They can map the relationship of the hazard to the true, unobserved trajectory of the longitudinal measurement^10^. The basic joint model (JM) allows this by estimating a linear mixed effects model and a relative risk model and coupling the submodels together by a joint likelihood^7^. Another variant is the joint latent class mixed model (JLCMM)^11,12^. This model can capture the possible heterogeneity of the population by finding latent classes of trajectories for longitudinal measurements associated with the risk of the event. The two submodels are tied together only via class membership.

Two recent T1D studies have implemented variants of joint models^13,14^. Recently, joint models were used to study the association between gluten intake and the risk of islet autoimmunity (IA)^15^. Otherwise, joint models have been little used in longitudinal nutritional data analysis so far^16,17^ even though they have a great potential. Joint models can (i) identify individual exposure trajectories even when exposure is observed only at some time points and may include missing values, (ii) smooth out measurement error and (iii) have the potential by JLCMMs to detect periods of sensitivity and risk groups.

First, we set out to assess the association between longitudinal consumption of meat, fish, and eggs during childhood and the risk of advanced IA and T1D in a population-based cohort of young children with HLA-DQB1-conferred susceptibility to T1D. Second, we provide a case investigation of whether associations can be more efficiently identified and more appropriately modeled with joint models (JM and JLCMM) than with the CRM.

## Material and Methods

### Subjects

This study is part of the Finnish prospective population-based Type 1 Diabetes Prediction and Prevention (DIPP) birth cohort study^18^. Newborn infants born in the Tampere and Oulu University Hospitals between September 1996 and September 2004 were screened for *HLA-DQB1*-conferred susceptibility to T1D using cord blood samples. Infants carrying increased genetic susceptibility (HLA-DQB1*02/0302 heterozygous and DQB1*0302/x-positive subjects [x stands for homozygosity or a neutral allele]) were monitored for diabetes-associated autoantibodies and growth up to the age of 15 years or until the manifestation of T1D. The DIPP Nutrition Study includes detailed measurements of childhood food consumption, with 6081 at-risk children (78% of invited children).

The study adheres to the Declaration of Helsinki. The following local ethics committees approved the study protocol: The ethical committee of University of Oulu, the Joint Commission on Ethics of the Turku University and the Turku University Hospital, the ethical committee of the city of Oulu, the ethical committee of the Pirkanmaa Hospital District, and the ethical committee of the University Hospital of Tampere. Parents gave their written informed consent for genetic testing of the newborn infant and for participation in the follow-up.

In the present analysis, the inclusion criteria was having at least one 3-day food record before the last autoantibody measurement, and having at least one autoantibody measurement and/or T1D diagnosis. Among the 5545 children who fulfilled the criteria the median number of autoantibody measurements was 14 (interquartile range, IQR: 6–18) and 3-day food records 5 (IQR: 3–8) per child. Of the 5545 children, 5506 were eligible for the energy-adjusted analyses. The excluded 39 children had no growth data for the first year of life, and therefore calculation of total energy intake was not possible for them during that period. Furthermore, as they had no food record data after the first year, they could not be included to the energy-adjusted analyses. The data used in the present analysis were updated in 2017.

### Dietary assessment

Three-day food records were collected at the age of 3 and 6 months and at 1, 2, 3, 4, and 6 years^19^. For some children, records were also collected at the age of 5 years although it was not the scheduled measurement point. Families recorded all foods and drinks the child had consumed with amount, brand, recipe, and preparation method. Portion size was estimated either with household measures or by using a food portion picture booklet. Trained study nurses checked the food records and a trained nutritionist entered the data^19^.

The data entry and calculation of intakes of meat, fish, and eggs were done with in-house software by using an annually updated national food composition database^20^. The definition of meat includes red meat, viscera, poultry, meat products, and sausages. Fish includes fish, fish products, and seafood. The food composition database and connected software enabled the summarization of the intake of each food from different food items, for example, the amount of eggs coming from a cake. The dietary data were used up to the detection of advanced IA.

Total energy intake was calculated based on food records and breastfeeding. For those who were breastfed, total energy intake was estimated based on age, bodyweight, and expected energy deposition needed for growth^21^.

### Laboratory methods

Of the four T1D–associated autoantibodies analyzed, islet cell antibodies (ICA) were used as the primary screening tool. When a child seroconverted to positivity for ICA for the first time, all of the child’ s preceding (starting from birth) and subsequent samples were analyzed for insulin autoantibodies (IAA), glutamic acid decarboxylase antibodies (GADA), and islet antigen 2 antibodies (IA-2A). The ICAs were quantified by a standard indirect immunofluorescence method and IAA, GADA, and IA-2A with specific radiobinding assays as described previously^22^. Transplacentally transferred autoantibodies were excluded from the analyses. The endpoint of advanced IA was defined as repeated positivity for ICA together with at least one other diabetes-associated autoantibody (IAA, GADA or IA-2A), or T1D diagnosis. Additional analyses were performed with T1D alone as an endpoint. Data of T1D diagnosis was obtained from the national pediatric diabetes registry.

### Sociodemographic and perinatal characteristics

Information on each child’s sex, maternal vocational education, and diabetes in a first-degree relative (i.e., familial diabetes) was collected with a structured questionnaire completed by parents after the birth of the child. Information on perinatal characteristics was received from the Medical Birth Registry.

### Statistical analysis

We used and evaluated the performance of three different statistical models for studying the association between the absolute daily consumption of meat, fish, and eggs, and the risk of advanced IA. The models were the CRM, in which the longitudinal consumption was used as a piecewise constant step function, the JM with a current value association structure, and the JLCMM. Multiply imputed mean food intakes of the 3-day periods were used in the CRMs^23^, whereas in the joint models daily intakes were used. Times-to-event for children with advanced IA were set to the middle of the time interval between the last advanced IA-negative and the advanced IA-positive (including T1D diagnosis) measurement. JMs for all the foods and JLCMM for fish were also ran with T1D to investigate consistency between alternative endpoints.

Each of the models was fitted as an univariate model with consumption only and as an adjusted model incorporating important confounding factors identified by a stepwise selection strategy^6^. In addition, CRMs and JMs were fitted as energy-adjusted models with consumption relative to total energy intake. In the energy-adjusted models, mean food intakes in grams (g) were divided by mean total energy intakes in megajoules (MJ) over the 3-day period for CRM, and daily food intakes were divided by daily total energy intakes for JM. The selected confounding factors were sex of the child, genetic risk of the child and familial diabetes. Maternal vocational education was not included in the final models, because the number of children with advanced IA was very small for some of the factor value combinations. The confounders were used in the survival parts of the models.

We assume that the reader is familiar with the well-known CRM, but we briefly describe the JM and the JLCMM^7^. We then describe the implementation of submodels for food trajectories and our sensitivity analyses. Analyses were implemented with R, by using the jointModel function from the JM package^24^, Jointlcmm function from the lcmm package^25^, and the coxph function from the survival package.

#### The basic joint model

A JM consists of two submodels fitted simultaneously: a linear mixed effects model and a relative risk model, in which the hazard depends on the modeled food consumption. Let *m*_*i*_(*t*) denote the true and unobserved time trajectory of the food consumption and let *y*_*i*_(*t*) denote the observed food consumption for child *i* at time *t*.

The two submodels were of the form:

1 $$\{\begin{array}{l}{y}_{i}(t)={m}_{i}(t)+{\varepsilon }_{i}(t)={\beta }_{0}+{b}_{0i}+\sum _{k=1}^{5}\,({\beta }_{k}+{b}_{ki}){B}_{k}(t)+{\varepsilon }_{i}(t),\\ {h}_{i}(t|{M}_{i}(t),{w}_{i})=\exp \{{\gamma }^{T}{w}_{i}+\alpha {m}_{i}(t)\}{h}_{0}(t),\end{array}$$

where *β*_0_ and *b*_0*i*_ denote the fixed part and subject-specific random part of the intercepts, respectively, and *β*_*k*_ and *b*_*ki*_ denote the fixed effects and subject-specific random effects parts of the regression parameters, respectively. The covariance matrix of the normally distributed random effects was diagonal. *B*_*k*_(*t*) denotes the value of *k* th B-spline basis function for a piecewise cubic polynomial spline at time *t*, and *ε*_*i*_(*t*) are normally distributed independent errors with mean 0 and variance *σ*^2^. In the survival model ${M}_{i}(t)=\{{m}_{i}(s),0\le s < t\}$ denotes the history of the longitudinal process until *t*, and *w*_*i*_ denotes a vector of baseline covariates with a corresponding vector of regression parameters *γ*. The submodels are combined via the covariate *m*_*i*_(*t*), and *α* denotes the corresponding regression parameter. The *h*_0_(*t*) denotes the baseline hazard at time *t* which was set as a piecewise constant with knots at the ages of 1.99 and 3.99^26–28^. Based on the basic structure of the JM, the time-to-event data were used only up to the last point of food consumption measurement (6 years). To enable comparison, the same time period was used in the CRMs.

#### Joint latent class mixed model

A JLCMM consists of three submodels: the linear mixed effects model, the relative risk model and a multinomial logistic model for the latent class probabilities. The model assumes the food trajectories and hazards arise from several underlying classes, and each child belongs to each particular class with some probability. Compared to a JM, latent class -specific parameters were added to the longitudinal submodel, and the baseline hazard in the survival submodel was set as class-specific with an additional knot at 5.99 years. The two submodels were connected via class membership. Standard errors of piecewise hazard ratios were obtained with the delta method, and the overall hazard ratio was obtained with a JLCMM assuming baseline hazards to be proportional over the entire time period. The effects of the baseline covariates were assumed to be the same across classes. The multinomial logistic regression submodel included the intercept only to ensure that class membership probabilities were based solely on the food consumption profiles.

The submodels were of the form:

2 $$\{\begin{array}{rcl}{y}_{i}(t|{c}_{i}=g) & = & {\beta }_{0g}+{b}_{0ig}+\sum _{k=1}^{5}\,({\beta }_{kg}+{b}_{kig}){B}_{k}(t)+{\varepsilon }_{i}(t),\\ {h}_{i}(t|{c}_{i}=g) & = & \exp \{{\gamma }^{T}{w}_{i}\}{h}_{0g}(t),\\ Pr({c}_{i}=g) & = & \exp \{{\lambda }_{g}\}/\sum _{l=1}^{G}\,\exp \{{\lambda }_{l}\},\end{array}$$

where *g* = 1, …, *G* denotes the latent class, and *λ*_*g*_ defines class membership probabilities. The submodels are combined by the class indicator *c*_*i*_. The time-to-event data were used up to the age of 15 years. In the JLCMM, detection of underlying classes, their distinct trajectories and inference on the parameters of the class-specific hazards are of interest.

Choosing the number of latent classes is a central issue in JLCMMs, which is often based on the Bayesian information criterion (BIC)^29,30^. It has also been recommended that the choice of the number of latent classes should, besides the BIC, be based on a good discrimination between classes, correct predictions, satisfactory conditional independence and meaningful latent classes, in order to use the solution with the fewest classes that provides a satisfactory fit to the data^11,12,31^. Therefore, we fitted the models with 1–4 latent classes (when converged) for each food item, and based the decision on the recommendations^32^. BIC curves for the JLCMMs can be found as Supplementary Fig. S1.

#### Spline-based food trajectory modeling

In the longitudinal submodels of the joint models we used flexible piecewise cubic polynomial spline functions to allow flexible individual food trajectories. B-spline cubic basis functions^33^ were used and the number of knots was set to *q* = 2 after finding a balance that allowed sufficient flexibility and avoided overfitting. To fully reach the potential flexibility of splines, knots were not placed equally or only on the measuring points^34,35^. We considered all four equispaced knot locations between each measurement^36^ and defined that there must be at least 2 measurements before the first knot, after the second knot, and between the knots. Knot searching was done by fitting linear mixed effects models with piecewise cubic polynomial splines using all knot combinations satisfying the criteria, and selection was done based on BIC. With the chosen criteria, knots were located in the middle parts of the age range and reasonably far apart, resulting in pleasing fits.

#### Sensitivity analyses

Sensitivity of the results for the assignment of times-to-event for children with advanced IA was assessed by running a multiple imputation approach to interval-censored data^37,38^. We also verified standard error estimates for the baseline hazards in the JLCMM for fish consumption with the delete-5 jackknife^39^. To investigate the robustness of the results to the choices of the number and the places of the knots in the baseline hazard, unadjusted JMs for meat, fish and eggs and unadjusted JLCMM for fish were ran with one knot removed and one additional knot, with each time interval including the same number of events. The sensitivity analyses were performed for the models with the advanced IA endpoint.

## Results

### Baseline characteristics and their association with the risk of advanced IA and T1D

Among the 5545 children with increased genetic risk of T1D, a total of 348 (6.3%) reached the endpoint of advanced IA during the first 15 years of life at a median age of 3.5 years (IQR: 1.8–6.6). Of them, 246 (4.4%) reached it before the age of 6 years at a median age of 2.5 years (IQR: 1.3–3.6). By the age of 15 years, 43 got T1D diagnosis before the autoantibody criterion was fulfilled and by the age of 6 years 34. A total of 195 (3.5%) progressed to T1D during the first 15 years of life at a median age of 6.4 years (IQR: 4.2–10.0) and 88 (1.6%) before the age of 6 years. Of the children, 92% were followed for autoantibodies up to 1 year, 84% up to 2 years, 68% up to 6 years and 33% up to 15 years of age. The median follow-up time was 10 years (IQR: 3.1–14.9). Boys, children with high *HLA*-*DQBI*-conferred risk, and children with a diabetic first-degree relative were at higher risk of advanced IA and T1D (Table 1). Maternal vocational education was associated with a child’s lower risk of advanced IA but the association was not so strong with T1D (Table 1). Genetic risk of the child and familial diabetes were not highly associated (see Supplementary Table S1). Kaplan-Meier curves (overall, and by sex, genetic risk and familial diabetes) for advanced IA can be found as Supplementary Fig. S2.

**Table 1:** Baseline characteristics of the children, and their association to the advanced islet autoimmunity (IA) and type 1 diabetes (T1D) based on the 15-year follow-up.

Characteristic	N (%)	Advanced islet autoimmunity			Type 1 diabetes		
		n (%)	HR (95% CIs)^1^	P	n (%)	HR (95% CIs)^1^	P
**Sex of the child**							
Boy	2950 (53.2)	212 (7.2)	1		115 (3.9)	1	
Girl	2595 (46.8)	136 (5.2)	0.72 (0.56, 0.93)	0.012	80 (3.1)	0.76 (0.57, 1.01)	0.062
**Genetic risk**							
Moderate (DQB1*0302/x)^2^	4457 (80.4)	228 (5.1)	1		123 (2.8)	1	
High (DQB1*02/0302)	1088 (19.6)	120 (11.0)	1.95 (1.49, 2.55)	<0.001	72 (6.6)	2.49 (1.86, 3.33)	<0.001
**Familial diabetes**							
No	5001 (90.2)	299 (6.0)	1		161 (3.2)	1	
Yes	329 (5.9)	41 (12.5)	2.13 (1.45, 3.12)	<0.001	29 (8.8)	2.87 (1.93, 4.27)	<0.001
Missing information	215 (3.9)	8 (3.7)	0.30 (0.10, 0.92)	0.036	5 (2.3)	0.30 (0.09, 1.04)	0.057
**Mother’s vocational education**							
None	354 (6.4)	33 (9.3)	1		17 (4.8)	1	
Vocational school or course	1465 (26.4)	81 (5.5)	0.45 (0.29, 0.72)	<0.001	40 (2.7)	0.55 (0.31, 0.96)	0.037
Secondary vocational education	2357 (42.5)	137 (5.8)	0.38 (0.25, 0.59)	<0.001	74 (3.1)	0.63 (0.37, 1.07)	0.088
University studies or degree	1210 (21.8)	88 (7.3)	0.57 (0.36, 0.89)	0.014	57 (4.7)	1.00 (0.58, 1.71)	0.991
Missing information	159 (2.9)	9 (5.7)	1.58 (0.55, 4.47)	0.393	7 (4.4)	2.47 (0.79, 7.67)	0.119

^1^Estimates are hazard ratios from the Cox proportional hazards model including all the four baseline factors in the table.

^2^*x* not equal to *02, *0301, or *0602.

### Consumption of meat, fish and eggs

**Figure 1:**
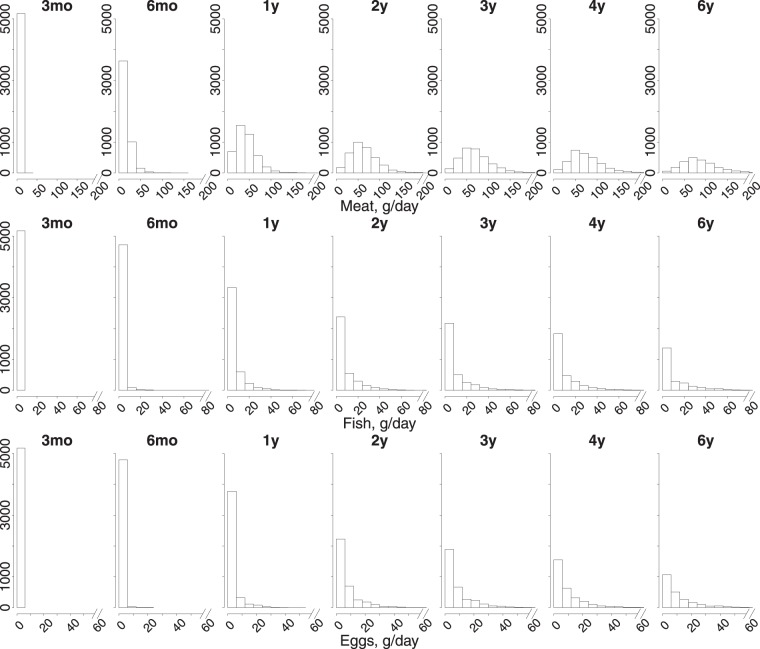
The distribution of daily consumption of meat and meat products, fish and fish products and eggs in grams by age from 3-day food records.

At 3 months of age the selected foods had not been consumed at all. The consumption of meat, fish and egg increased by age. At 6 years, 99.4% of children with completed 3-day food record reported the use of meat, 49.8% reported that of fish and 91.9% reported that of eggs with mean consumption of 88 g/day (SD: 61), 11 g/day (SD: 27) and 10 g/day (SD: 19), respectively. The distributions of the average daily consumption of the foods by age from the 3-day food records are presented in Fig. 1.

### Food consumption and the risk of advanced IA and T1D

Based on the JM, the consumption of meat was marginally associated with advanced IA: the hazard ratio was 1.05 (95% CI: 0.99, 1.12) per 10 g increment in the consumption in the model adjusted for selected confounding factors, and the energy-adjusted hazard ratio was 1.04 (95% CI: 1.00, 1.07) per 1 g/MJ increment in the consumption (Table 2). The results with T1D as an endpoint were consistent with these results (Table 3). The CRM, however, did not reveal this association although estimates from both models were consistent in direction. The JM gave slightly wider confidence intervals than the CRM. Perceptible latent classes for meat consumption and the risk of advanced IA were not identified with the JLCMM.

**Table 2:** Risk of advanced islet autoimmunity (IA) associated with the consumption of meat and meat products, fish and fish products and eggs based on the Cox regression model (CRM) and the basic joint model (JM).

Food consumption	Unadjusted^1,2^	P	Adjusted^1,2,3^	P	Energy-adjusted^1,4^	P
**Cox regression model**						
Meat and meat products	1.01 (0.96, 1.05)	0.798	1.01 (0.96, 1.05)	0.831	1.00 (0.98, 1.02)	0.877
Fish and fish products	0.99 (0.86, 1.13)	0.840	0.99 (0.86, 1.14)	0.838	0.99 (0.94, 1.05)	0.815
Eggs	1.08 (0.91, 1.28)	0.385	1.08 (0.91, 1.29)	0.373	1.04 (0.96, 1.12)	0.379
**Basic joint model**						
Meat and meat products	1.06 (1.00, 1.12)	0.054	1.05 (0.99, 1.12)	0.091	1.04 (1.00, 1.07)	0.050
Fish and fish products	1.14 (0.91, 1.43)	0.261	1.12 (0.89, 1.41)	0.318	1.09 (0.95, 1.24)	0.210
Eggs	1.22 (0.94, 1.57)	0.132	1.23 (0.95, 1.59)	0.119	1.16 (1.01, 1.33)	0.031

^1^Values are hazard ratios with 95% CIs in parentheses.

^2^Per 10 grams increment in the consumption of the particular food.

^3^Models adjusted for sex of the child, genetic risk of the child and familial diabetes.

^4^Per 1 gram/megajoule increment in the consumption of the particular food.

**Table 3:** Risk of type 1 diabetes (T1D) associated with the consumption of meat and meat products, fish and fish products and eggs based on the basic joint model (JM).

Food consumption	Unadjusted^1,2^	P	Adjusted^1,2,3^	P	Energy-adjusted^1,4^	P
Meat and meat products	1.13 (1.03, 1.24)	0.011	1.13 (1.02, 1.24)	0.015	1.09 (1.02, 1.17)	0.010
Fish and fish products	1.20 (0.85, 1.70)	0.296	1.18 (0.83, 1.68)	0.354	1.11 (0.87, 1.42)	0.398
Eggs	0.64 (0.24, 1.71)	0.377	0.67 (0.26, 1.73)	0.409	0.74 (0.34, 1.63)	0.458

^1^Values are hazard ratios with 95% CIs in parentheses.

^2^Per 10 grams increment in the consumption of the particular food.

^3^Models adjusted for sex of the child, genetic risk of the child and familial diabetes.

^4^Per 1 gram/megajoule increment in the consumption of the particular food.

**Figure 2:**
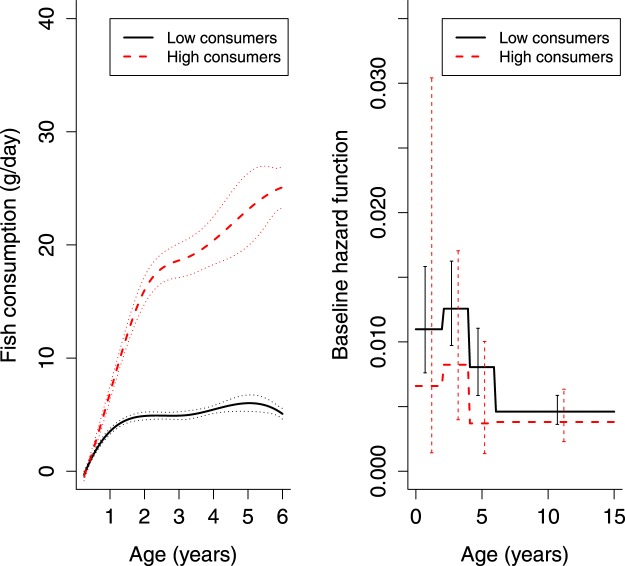
Two latent classes with different fish consumption trajectories (with 2.5% and 97.5% percentiles of the Monte Carlo approximation of the posterior distribution), and their associated baseline hazards (with 95% CIs) of advanced islet autoimmunity (IA).

**Figure 3:**
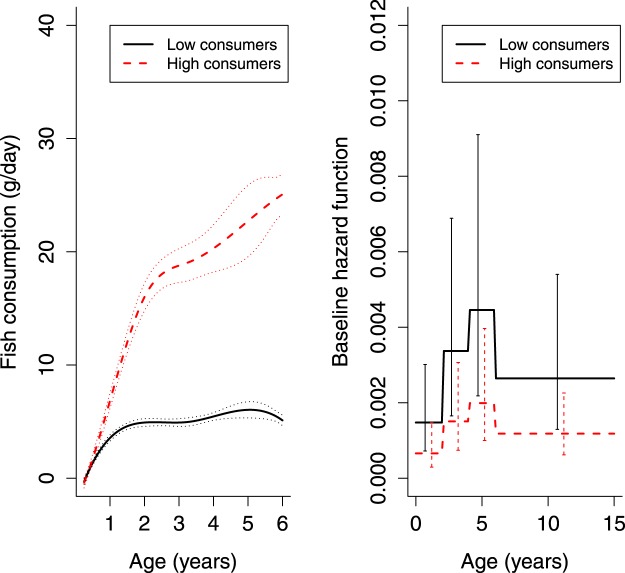
Two latent classes with different fish consumption trajectories (with 2.5% and 97.5% percentiles of the Monte Carlo approximation of the posterior distribution) and their associated baseline hazards (with 95% CIs) of type 1 diabetes (T1D), based on the joint latent class mixed model (JLCMM) assuming baseline hazards of the latent classes to be proportional over the entire time period.

The consumption of fish was not associated with advanced IA in either the JM or the CRM (Table 2), or with T1D in the JM (Table 3). The hazard ratio estimates from the JM and CRM were not consistent in direction but their confidence intervals were not mutually exclusive. However, JLCMM found two perceptible latent classes in the consumption trajectories of fish and the risk of advanced IA (Fig. 2). Similar latent classes were found with the T1D endpoint (Fig. 3).

Low fish-consumer class included children consuming very little fish: the approximate consumption of 5 g/day over the entire time period starting from the age of 1 year. High fish-consumer class showed a steep age gradient in fish consumption from no consumption to an average of 25 g/day at 6 years. The risk of advanced IA among high fish consumers tended to be lower over the entire period compared to low fish consumers. Piecewise hazard ratios were consistently below one, but none of them alone were significant (Table 4). The number of children with advanced IA was small among high fish consumers in some intervals, which resulted in extremely large standard errors both from model-based and jackknife-based estimation. The overall hazard ratio of advanced IA for high fish consumers, compared to low fish consumers, was 0.68 (95% CI: 0.44, 1.05) (Table 4) and of T1D 0.45 (95% CI: 0.23, 0.86) in the models adjusted for selected confounding factors. The number of the children with T1D was 15 (1.6%, N = 941) among the high fish consumers and 180 (3.9%, N = 4604) among the low consumers. The distributions of the confounding factors in the two latent classes of the fish consumption and the risk of advanced IA can be found as Supplementary Table S2 and the average consumption of meat and eggs in the two latent classes as Supplementary Fig. S3. The Kaplan-Meier curves for latent classes can be found as Supplementary Fig. S4. We also performed Fisher’ s exact test as in^30^ for the number of children with advanced IA by the obtained grouping, which signaled an association (*P* < 0.001).

**Table 4:** The results of the joint latent class mixed model (JLCMM) for fish and fish products consumption: The number of the children reaching the endpoint of advanced islet autoimmunity (IA), and piecewise and overall hazard ratios with 95% CIs for the baseline hazard of high fish consumers in relation to the baseline hazard of low fish consumers.

Age interval	Children with advanced IA		Unadjusted^2^	P	Adjusted^2,3^	P
	Low consumers N = 4619^1^ (83.3%)	High consumers N = 926^1^ (16.7%)				
0–2^4^	96	3	0.60 (0.10, 3.73)		0.62 (0.11, 3.67)	
2–4^4^	87	9	0.66 (0.28, 1.55)		0.65 (0.27, 1.53)	
4–6^4^	46	5	0.46 (0.15, 1.41)		0.45 (0.15, 1.38)	
6–15^4^	84	18	0.83 (0.45, 1.53)		0.81 (0.44, 1.49)	
overall^5^	313 (6.8%)	35 (3.8%)	0.69 (0.45, 1.05)	0.085	0.68 (0.44, 1.05)	0.082

^1^Number of the children is for the unadjusted model, similar in the adjusted model.

^2^Values are hazard ratios with 95% CIs in parentheses.

^3^Model adjusted for sex of the child, genetic risk of the child and familial diabetes.

^4^Estimates of standard errors obtained with delta method.

^5^Estimates and P-values obtained from the joint latent class mixed model assuming baseline hazards of the latent classes to be proportional over the entire time period.

The CRM or JM did not suggest consistent associations between egg consumption and advanced IA although the energy-adjusted hazard ratio signaled a marginally increased risk association (Table 2). However, the hazard ratio estimates from the JM and CRM were consistent in direction. The estimate from the CRM was closer to unity compared to the JM, and the JM gave wider confidence intervals. The results with T1D as an endpoint did not suggest any associations either, and the hazard ratio estimates were not consistent in direction with the advanced IA results (Table 3). Perceptible latent classes for egg consumption and the risk of advanced IA were not identified with JLCMM.

### Sensitivity analyses

Adjustment of the models for sex, genetic risk and familial diabetes did not change the estimates substantially in any of the models and results were not sensitive to energy adjustment, regardless of the outcome. Estimates of the hazard ratios were not sensitive to interval censoring although their standard errors increased a little after the multiple imputation approach. The choices of the number and the places of the knots in the baseline hazard had little effect on the hazard ratio estimates but did not change the interpretation of the results. Reducing the number of the knots slightly weakened, and adding slightly strengthened the findings. For confounding factors, hazard ratios with 95% CIs from the adjusted joint models with the advanced IA endpoint are presented as Supplementary Table S3.

## Discussion

We examined the association between the early consumption of meat, fish, and eggs, and the risk of advanced IA in children with increased *HLA*-conferred genetic susceptibility to T1D by using a CRM, JM, and JLCMM. The associations of these foods to the risk of T1D were also studied by using the JM and JLCMM. The JM suggested marginal association between higher meat consumption and increased risk of advanced IA, but this could not be verified with a CRM. The JLCMM suggested a group of children with high fish consumption profile, which was marginally associated with lower risk of advanced IA. This association was not identified by the JM or the CRM. The higher meat consumption was also associated to the higher risk of T1D based on the JM, and the JLCMM suggested the similar fish-consumer classes as with the advanced IA endpoint, with a protective association of high fish consumption to the risk of T1D. No consistent evidence of an association between egg consumption and the risk of advanced IA or T1D was found.

The major strengths of the study are a large study population and a long follow-up that together enabled great statistical power; in addition, the collection of dietary information took place before the development of advanced IA excluding reporting bias. From a methodological viewpoint, use of different statistical models enabled a comparison and brought different perspectives to the content.

The major limitations of the study are that the study population consisted of at-risk children, and it is unclear whether our epidemiological findings apply to the general population. Additionally, we were only able to investigate the foods separately. Simultaneous investigation could bring further insight by identification of additive and interaction effects. However, software for joint models for multivariate longitudinal data is still lacking^40^.

The CRM has previously been found to be far too rough in the modeling of association between a longitudinal exposure and time-to-event endpoint, and to have a tendency to underestimate the association parameter^41,42^. Our results support these findings as the estimates from the CRMs were all attenuated towards unity compared to the JM. The JM gave wider confidence intervals than the CRM mostly based on its ability to take within individual day-to-day variation of food consumption into account. Another major advantage of the joint models compared to the CRM is the built-in handling of incomplete data. The computational burden and the convergence problems might be the practical limitations of the wider use of joint models, particularly with JLCMMs. However, the marginal associations would have not been found without the use of joint models.

Despite the lack of evidence of an association between fish intake and risk of advanced IA or T1D based on the CRM or JM, the JLCMM revealed groups with higher intake of fish and marginally lower risk of advanced IA and T1D. A relatively small intake of fish might be the reason that an association was not observed when the exposure was considered as a continuous one in the JM. Instead, the sufficient use of fish might be important considering the advanced IA and T1D risk. The children in the high fish-consumer JLCMM-classes used fish on average 70 g/week at 1 year with an increasing pattern to 175 g/week at 6 years (Fig. 2). Taking into consideration the portion sizes, the classes identified children who complied with the Finnish dietary recommendations for children^43^ which instruct to eat fish 2–3 times a week. The marginal finding of higher fish consumption being associated with lower risk of advanced IA supports two previous prospective observations of a protective association between fish-derived fatty acid status and IA development^44,45^. The potential benefits of fish consumption may be related to the n-3 fatty acids, which play role in the development and function of the immune system and inflammatory reactions^46^.

Child’s meat consumption has not been related to advanced IA or T1D development in any prospective setting. Maternal meat consumption during lactation was associated with child’s increased risk of T1D in a prospective cohort^27^. One case-control study and an ecological correlational analysis suggest that meat consumption is associated with increased risk of TID^47,48^. The potential mechanism of action of high meat consumption is not yet known. Findings relating advanced glycosylation end product and nitrite or N-nitroso compound intake to the disease process led to interest in processed meat products^49–51^ in the disease etiology. Also, heme iron and proteins of meat may play a role^52^. Human gut microbiota may have an important role in the development of T1D and meat consumption is known to affect the gut microbiota^52,53^.

Together with other known health-benefits of fish and fish-derived fats and the health risks related to high meat consumption, our results are in line with the current Finnish dietary recommendations^43^. Future prospective studies should repeat the analyses. The role of fish and fish oil consumption in the prevention of T1D could be clarified in a trial setting.

In conclusion, our findings from this study suggest that a child’s intake of meat might be directly, and fish inversely, related to the development of advanced IA and T1D. Disease hazards in longitudinal nutritional epidemiology are more appropriately and efficiently modeled by joint models than with naive approaches.

## Supplementary information

Supplementary information for A Joint Modeling Approach for Childhood Meat, Fish and Egg Consumption and the Risk of Advanced Islet Autoimmunity

## Data Availability

Data supporting the findings of this study are available from the corresponding author on reasonable request.
