# Supplementary material for: A Joint Modeling Approach for Childhood Meat, Fish and Egg Consumption and the Risk of Advanced Islet Autoimmunity
Source: Sci Rep. 2019 May 23;9:7760. doi: 10.1038/s41598-019-44196-1 (PMC6533366; doi:10.1038/s41598-019-44196-1)
Supplement: Supplementary file 1 — Supplementary information for A Joint Modeling Approach for Childhood Meat, Fish and Egg Consumption and the Risk of Advanced Islet Autoimmunity [file 41598_2019_44196_MOESM1_ESM.pdf]

Supplementary information for "A Joint Modeling Approach for Childhood Meat, Fish and Egg Consumption and the Risk of Advanced Islet Autoimmunity"

Essi Syrjälä, Jaakko Nevalainen, Jaakko Peltonen, Hanna-Mari Takkinen, Leena Hakola, Mari Åkerlund, Riitta Veijola, Jorma Ilonen, Jorma Toppari, Mikael Knip, Suvi M. Virtanen

Supplementary Table S 1: Frequency table of the genetic risk and familial diabetes, Fisher's exact test P=0.283.

		Genetic risk	
		Moderate	High
Familial diabetes	No	4027 (80.5)	974 (19.5)
	Yes	257 (78.1)	72 (21.9)

Supplementary Table S 2: Distributions of the counfounding factors in the two latent classes of fish consumption and the risk of advanced islet autoimmunity (IA) based on the adjusted joint latent class mixed model (JLCMM).

	Low consumers[1] N=4619	High consumers[1] N=926
Sex of the child		
Boy	2447 (53.0)	503 (54.1)
Girl	2169 (47.0)	426 (45.9)
Genetic risk		
Moderate	3700 (80.2)	757 (81.5)
High	916 (19.8)	172 (18.5)
Familial diabetes		
No	4143 (93.8)	858 (94.1)
Yes	275 (6.2)	54 (5.9)
Mother's vocational education		
None	318 (6.9)	36 (3.9)
Vocational school or course	1268 (27.5)	197 (21.2)
Secondary vocational education	1941 (42.0)	416 (44.8)
University studies or degree	941 (20.4)	269 (29.0)
Missing information	148 (3.2)	11 (1.2)

[1]Values are class frequencies with proportions in the parentheses

Supplementary Table S 3: Hazard ratios with 95% CIs of the counfounding factors associated with the advanced islet autoimmunity (IA) from the basic joint models (JMs) and from the joint latent class mixed model (JLCMM).

Characteristic	JM meat	JM fish	JM egg	JLCMM fish
Sex of the child				
Boy	1	1	1	1
Girl	0.74 (0.58, 0.96)	0.73 (0.57, 0.94)	0.73 (0.57, 0.94)	0.70 (0.57, 0.87)
Genetic risk				
Moderate	1	1	1	1
High	1.93 (1.48, 2.52)	1.92 (1.47, 2.51)	1.92 (1.47, 2.51)	2.19 (1.76, 2.73)
Familial diabetes				
No	1	1	1	1
Yes	2.11 (1.44, 3.09)	2.12 (1.44, 3.10)	2.13 (1.46, 3.13)	2.02 (1.46, 2.81)
Missing information	0.71 (0.32, 1.61)	0.72 (0.32, 1.62)	0.72 (0.32, 1.61)	0.69 (0.35, 1.35)

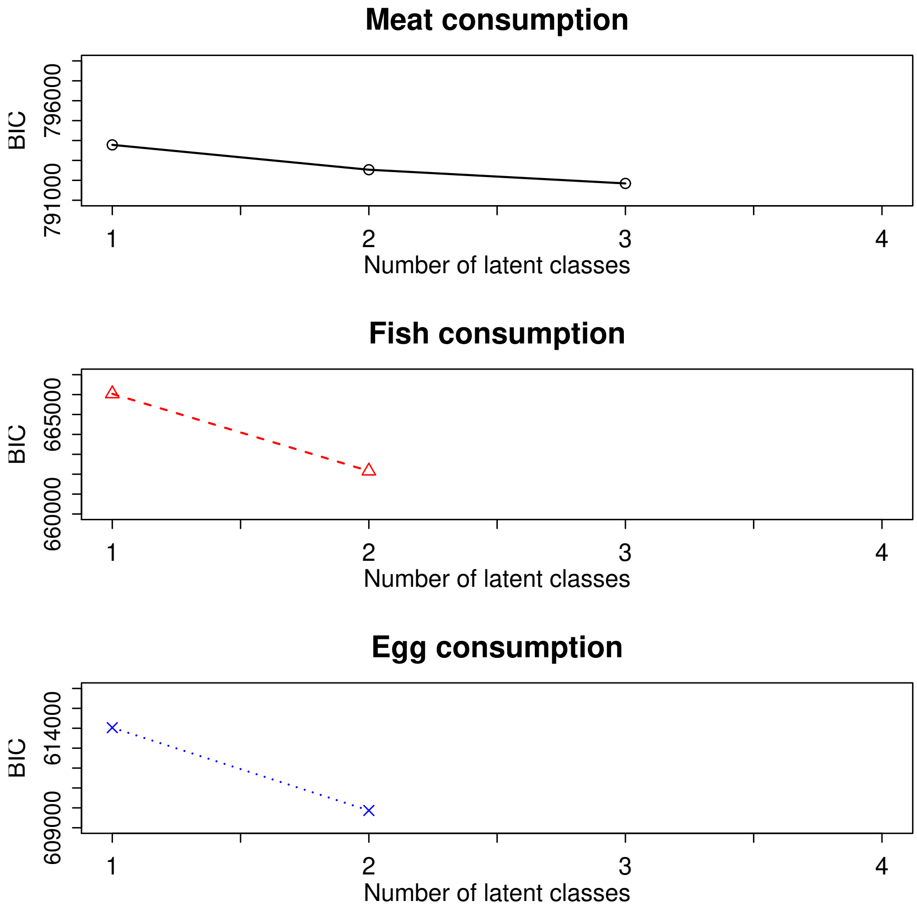

Supplementary Figure S 1: BIC curves for converged joint latent class mixed models with different number of latent classes for the consumption of meat, fish and eggs, and the risk of advanced islet autoimmunity (IA).

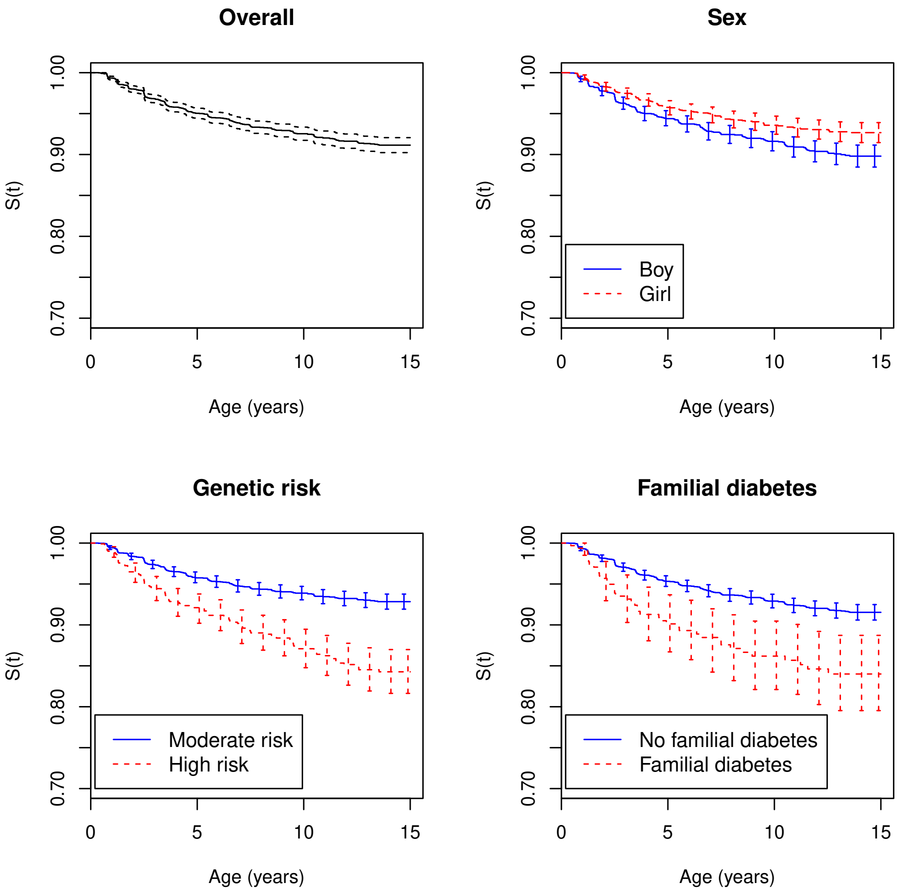

Supplementary Figure S 2: Kaplan-Meier curves with 95% CIs for advanced islet autoimmunity (IA): Overall curve and curves by sex of the child, genetic risk of the child and familial diabetes.

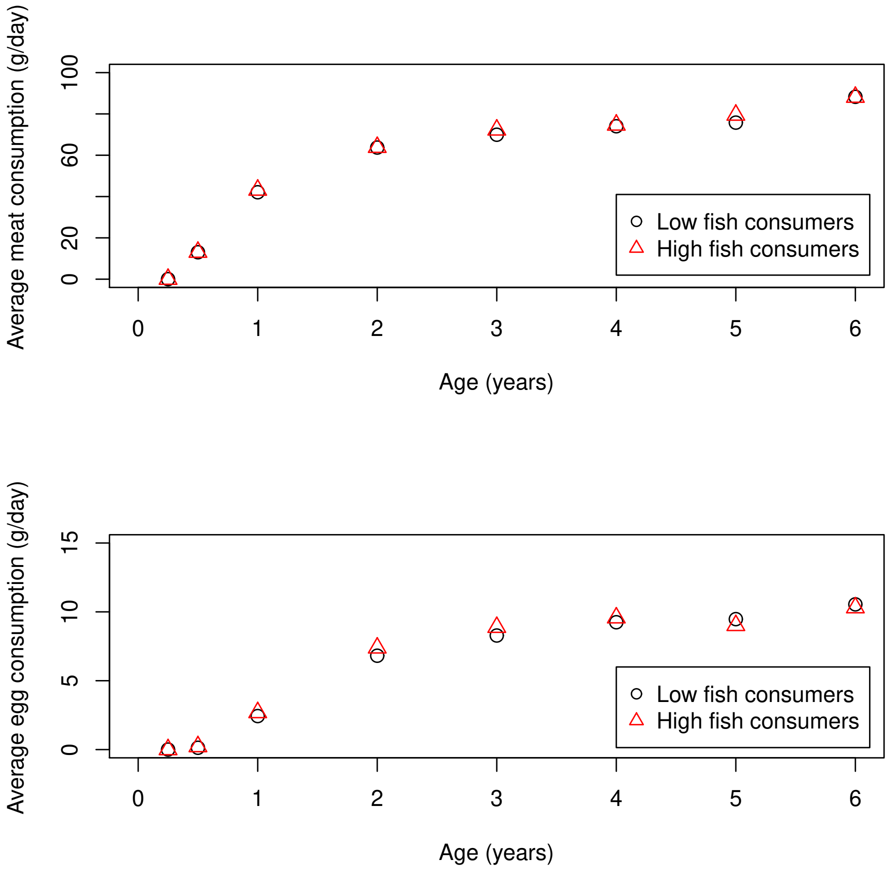

Supplementary Figure S 3: Average consumption of meat and eggs at the different ages in the two latent classes of fish consumption and the risk of advanced islet autoimmunity (IA) based on the adjusted joint latent class mixed model (JLCMM).

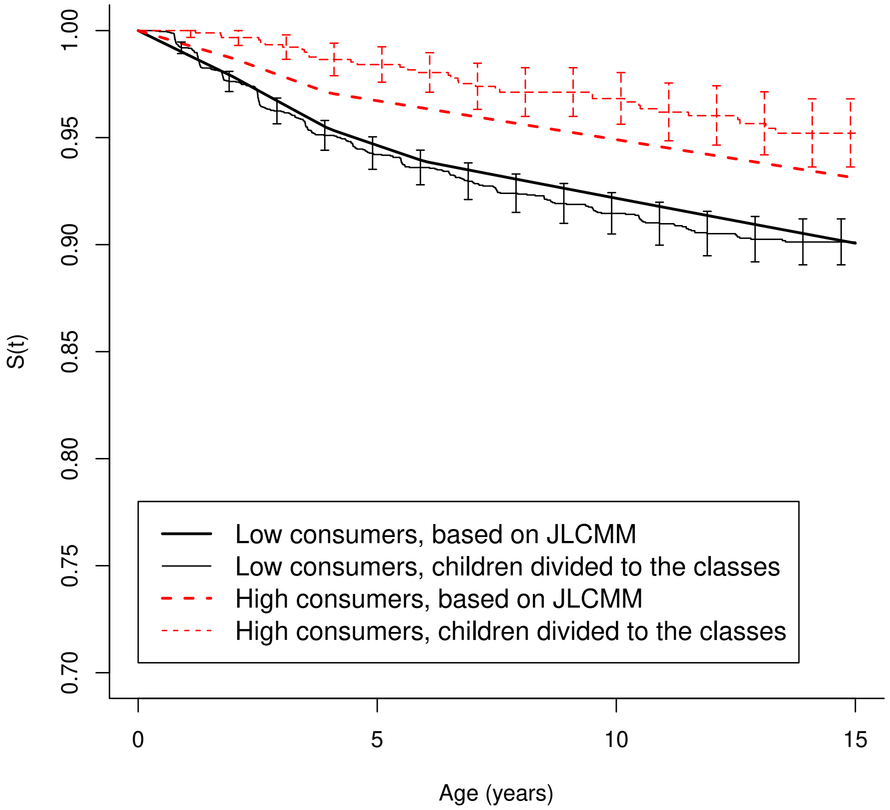

Supplementary Figure S 4: Kaplan-Meier curves for advanced islet autoimmunity (IA) by two latent classes of fish consumption based on the adjusted joint latent class mixed model (JLCMM). Model based advanced IA-free survival and crude estimates with hard assignment of children to the most probable class (with 95% CIs).
